# The Approach and Prevention of Cholera

**Published:** 1883-08

**Authors:** 


### The Approach and Prevention of Cholera.

It is now nearly three months since a few cases of Asiatic cholera were reported at Bombay, and of these no notice was taken by European governments. The disease has been raging, however, in a moderate degree among the coolies in the Brahmaputra valley since 1880. It was not until the last week of June that the cholera appeared at Damietta in Egypt, a place, by the way, entirely off the great Oriental route of travel, and it has now assumed alarming proportions in the whole Nile valley. In these last days of July the mortality for the whole country has been nearly 1,000 deaths daily, 500 being in Cairo alone. Besides this the disease has reached Shanghai and other Chinese cities, and within a day or two thirty-seven sporadic cases have been reported in Bombay. With such virulence as this it cannot be expected that other countries can resist the plague unless most stringent precautions are taken. The commerce with the East remains uninterrupted, and we are not surprised to hear that six cholera cases have reached Russian ports, several cases the London dockyards, and one the port of Havre. Naturally every effort is being made to ward off the pestilence, Russia and Germany quarantining six days, Italy twenty days, and France and Spain twenty-one days. England does not quarantine at all, but has adopted a code of regulations laid down by the local government board, requiring custom officers to take careful note of all vessels from abroad, and if suspicious cases are discovered, they are removed to pest houses on shore; the healthy passengers are allowed to land, and the ship after disinfection may proceed to its destination. The officer has power to detain the vessel twelve hours. The *Lancet*, in commenting upon this, says that if the disease finds its way into England in spite of these precautions, we may be certain that it would, as often before, equally evade any system of quarantine. In the United States the quarantine lasts five days, the sick being removed to floating or isolated hospitals.

Cholera has usually entered Europe by way of Russia. We find it frequently stated in the secular press, that the disease having appeared so late in Egypt, it is probable that it cannot progress much farther before cold weather comes in to put an end to it. Although cold weather does tend to moderate its progress it does not always check it. For instance in 1830 it spread over the whole of Russia, and the first cases reached England in July, 1831, from Riga; but it did not break out generally until October, when the monthly death rate was, for November, 97, gradually increasing to 1519 in March. Generally it is not until the year following its appearance in Oriental lands that it reaches Europe and invades America.

As to the method of communication of the disease, most authorities agree that the germs are conveyed to persons through drinking water rendered impure by drainage from localities where the evacuations of the infected have been deposited. As to direct contagion there seems to be no more than by ordinary typhoid fever, nurses, physicians or post mortem examiners seldom becoming its victims; the infective principle seems to lie in all the excretions, and these, through the ground, or by soiling clothes, rags and other articles, propagate the disease. In cholera an ounce of prevention is worth a world of cure. By isolation, by prompt destruction of excreta and all articles which have come into contact with a patient, by careful attention to the purity of drinking water and the drainage of the soil in city districts, there seems little reason why cholera should not be limited to its first victim.

America lies, as it were, between two fires. We are open to invasion on both the Atlantic and Pacific seaboards, from Europe and Asia. The vast amount of emigration, however, from the former continent renders the Atlantic our most vulnerable side, and it is to effective quarantining and thorough sanitary inspection here that we must look for safety. Vessels leaving England for America are inspected by American medical officers before starting, and the search is supposed to be carefully made by officers here upon their arrival. It remains to be seen how well our public health physicians at the ports, appointed by our usual corrupt political method, will perform their duties. It is possible that danger to themselves may waken in them an energy sufficient to do more than draw their salaries.

